# Impact of Systemic and Radiation Therapy on Survival of Primary Central Nervous System Lymphoma

**DOI:** 10.3390/cancers17040618

**Published:** 2025-02-12

**Authors:** James Robert Janopaul-Naylor, Jimmy S. Patel, Manali Rupji, Kimberly Bojanowski Hoang, Neal Sean McCall, David C. Qian, Madison Lee Shoaf, Shawn Kothari, Jeffrey J. Olson, Hui-Kuo G. Shu, Alfredo Voloschin, Jim Zhong, Stewart G. Neill, Bree Eaton

**Affiliations:** 1 Department of Radiation Oncology, Emory University, Atlanta, GA 30322, USAhgshu@emory.edu (H.-K.G.S.);; 2 Department of Radiation Oncology, Memorial Sloan Kettering Cancer Center, New York, NY 10065, USA; 3 Biostatistics Shared Resource, Winship Cancer Institute at Emory University, Atlanta, GA 30322, USA; 4 Department of Neurosurgery, Emory University, Atlanta, GA 30322, USA; 5 Department or Radiation Oncology, University of Pittsburgh Medical Center, Pittsburgh, PA 15213, USA; 6 Department or Radiation Oncology, MD Anderson Cancer Center, Houston, TX 77021, USA; 7 Department of Hematology and Medical Oncology, Emory University, Atlanta, GA 30322, USA; 8 Orlando Health Cancer Institute, Orlando, FL 32806, USA; 9 Department of Pathology, Emory University, Atlanta, GA 30322, USA

**Keywords:** PCNSL, rituximab, methotrexate, bulky disease, WBRT

## Abstract

**Introduction**: Treatment for primary central nervous system lymphoma (PCNSL) includes high-dose methotrexate (HD-MTX)-based systemic therapy. Multiple regimens exist with no clear standard of care. We evaluated the impact of different therapies on PCNSL outcomes at a single institution. **Materials and Methods**: A total of 95 consecutive patients with PCNSL from 2002 to 2021 were retrospectively reviewed. The overall survival (OS) and progression-free survival (PFS) were estimated by the Kaplan–Meier method. The log-rank test and univariate and multivariable Cox regression analysis were used to evaluate the relationship between clinicopathologic and treatment variables with outcomes. **Results**: Among the 62 patients treated with definitive systemic therapy, the median age was 58; 71% had a Karnofsky performance status > 70, 49% had a single lesion, 31% received HD-MTX alone, and 61% had HD-MTX + rituximab. The two-year OS and PFS were 64% (95% CI: 49.8–75.0%) and 49% (95% CI: 35.0–60.9%), respectively. On multivariable analysis, the completion of > six cycles of HD-MTX (HR 0.40; 95% CI: 0.21–0.76; *p* = 0.01) was associated with superior OS, while the use of rituximab was associated with inferior OS (HR 2.82; 95% CI: 1.37–5.83; *p* = 0.01). There were no significant associations between the OS and PFS with temozolomide, the extent of surgical resection, radiation, or the size or number of initial lesions (all *p* > 0.05). **Discussion**: Innovation is needed to improve the outcomes for patients with PCNSL.

## Simple Summary

The backbone of management for newly diagnosed PCNSL is high-dose methotrexate with additional systemic, intrathecal, or radiation therapies for induction or consolidation. Rituximab is commonly used as an adjunct to methotrexate despite no significant benefit in the prospective trials IELSG32 or HOVON 105/ALLG NHL 24. Concerningly, patients in our study had significantly lower rates of overall survival and progression-free survival when rituximab was used with methotrexate. Moreover, intensive surgery and whole-brain radiotherapy were not associated with improved outcomes, and patients with bulky disease had numerically, but not statistically, significantly worse outcomes. We demonstrate real-world evidence for worse outcomes with treatment intensification, particularly for patients off-trial, as well as unmet needs for patients with PCNSL.

## 1. Introduction

Primary central nervous system lymphoma (PCNSL) is a rare intracranial malignancy with increasing incidence with age, with up to 4.32 cases per 100,000 for people 70 years old or older [1]. PCNSL represents about 4% of intracranial neoplasms and 4–6% of extranodal lymphomas, with a median overall survival (OS) ranging from months [1,2] to over 8 years [3,4,5] depending on prognostic factors and treatment. Given the paucity of cases, prospective randomized data are limited, and a wide variety of systemic therapies and radiotherapy regimens are implemented for induction and salvage treatment. The backbone of most regimens is high-dose methotrexate—often defined as ≥3.5 g/m^2^ (HD-MTX) [6]. Trials have examined the incorporation of radiation [7], intrathecal (IT) chemotherapy [8,9], rituximab, temozolomide (TMZ) [10], stem cell transplants [11], or the combination of HD-MTX, cytarabine, rituximab, and thiotepa (MATRix) [12,13], all yielding mixed results. Furthermore, given the heterogeneity of patient selection and protocol management, trial results can have limited generalizability [14].

Rituximab is an effective agent for the treatment of systemic therapy lymphoma and is generally well tolerated, but its role in PCNSL is less clear. Two randomized trials failed to identify a statistically significant benefit with the addition of rituximab to different regimens. In the three-arm IELSG32 trial, patients were randomized to HD-MTX with cytarabine alone, with rituximab, or with rituximab and thiotepa. While the arm with rituximab and thiotepa was superior, the arm with the addition of rituximab alone was not significantly different than the control arm [12]. In the HOVON 105/ALLG NHL 24 trial, patients were randomized to MTX, carmustine, teniposide, and prednisone with or without rituximab [15]. Similarly, the study showed no significant difference between the arms. Given the uncertainty around the optimal management of PCNSL, we examined our institutional experience to examine the effect of systemic and intrathecal therapy regimens on outcomes.

## 2. Materials and Methods

This is a single-institution retrospective cohort study of consecutive patients with pathologically confirmed PCNSL from 2002 to 2021. The study design was approved by our institutional Clinical and Translational Research Committee and Institutional Review Board with a complete waiver of informed consent. We *a priori* decided to evaluate patient (age, Karnofsky performance status [KPS], HIV status, and solid organ transplant), tumor (CSF cytology, the volume of contrast-enhancing lesion[s], and the number of lesions), and treatment features (the number of cycles of HD-MTX [0–5 vs. ≥6], first-line chemotherapy agents [use of rituximab, TMZ, or other agents], the use of IT chemotherapy or whole-brain radiotherapy [WBRT], and the extent of surgical resection) as covariates. The size of contrast-enhancing disease was defined categorically using a cutoff of 14 cc, corresponding to a 3 cm diameter sphere, which is the typical size limit for single-fraction radiosurgery. The outcomes assessed from the date of biopsy included the overall survival (OS) and progression-free survival (PFS). Progression was defined according to the RANO criteria [16]. Survival analysis was performed on the cohort of patients who received any chemotherapy stratified by patients who had rituximab incorporated into the induction therapy and those that did not. A post hoc subgroup analysis was performed on the cohort of patients who received any chemotherapy stratified by patients who had rituximab incorporated into induction therapy and those that did not, as well as an analysis of the systemic therapy regimen (HD-MTX alone, HD-MTX with rituximab, or HD-MTX with rituximab and TMZ [RMT]), and the incorporation of the year of diagnosis into the multivariable analysis.

Descriptive statistics were generated for all of the covariates as frequencies and percentages for the categorical variables, and the median and range for the numerical covariates. Time-to-event outcomes were estimated using the Kaplan–Meier method and compared using log-rank tests. Median follow-up was estimated using the reverse Kaplan–Meier estimator. Univariate (UVA) Cox regression analysis was used to determine associations with the OS and PFS. Variables that were significant at an alpha of 0.2 were used for the multivariable analysis (MVA). A multivariable Cox regression analysis using the backward selection method was used to select the covariates. Chi-square tests or Fisher’s exact tests, as appropriate, were used to determine the differences in the covariates between the cohort of patients treated with different systemic therapies. Statistical analysis was performed using SAS 9.4 (SAS Institute Inc., Cary, NC, USA) macros [17], and statistical significance was defined *a priori* at the 0.05 level.

## 3. Results

### 3.1. Patient Characteristics

**Figure 1:**
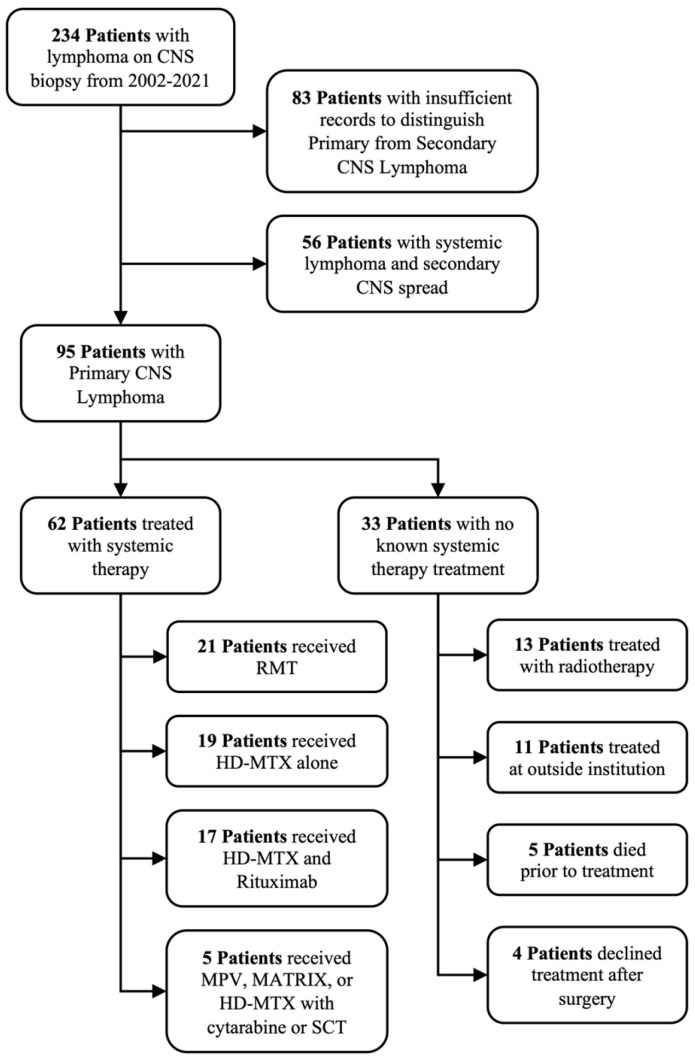
CONSORT diagram for patients with lymphoma on central nervous system (CNS) biopsy at our institution from 2002 to 2021. RMT is rituximab, high-dose methotrexate (HD-MTX), and temozolomide. SCT is stem cell transplant.

We identified 95 patients with pathologically confirmed PCNSL in the study period, 62 of which were treated with systemic therapy and were included in the primary analysis (Figure 1). At the time of biopsy (74.7%) or resection (26.3%), the median age was 58 (range 18–85) and the median KPS was 70 (range 20–100). In total, 10.5% of patients were HIV-positive, 4.2% had a solid organ transplant, and 91.6% had Diffuse Large B-cell histology. The remaining tumors were Marginal Zone (5.3%) or T-cell lymphomas (3.2%). Of the 60 patients with pre-treatment CSF cytological assessments, 18 (30.0%) had positive cytology. On staging MRI brain, the median number of intracranial lesions was 2 (range 1–22) and the median volume of contrast-enhancing disease was 12.6 cc (range 0.5–67.8 cc). Of the 70 patients with an evaluable MRI, there were 34 patients (48.6%) with single contrast-enhancing lesions at presentation. Patient and tumor characteristics are summarized in Table 1.

### 3.2. Treatment Characteristics

Of the entire 95 patients, 62 were treated with first-line systemic therapy, including three with upfront WBRT in addition to systemic therapy, either due to a poor initial performance status or a poor response to initial chemotherapy. Among the remaining 32 patients who did not receive systemic therapy, 13 patients were treated with radiotherapy alone. Four patients declined additional treatment after surgery, and five patients died prior to any treatment. Of the remaining 11 patients, 2 patients had an unknown dose of WBRT at an outside institution, and 9 patients elected to pursue unknown treatment elsewhere after follow-up with our institution for a median of 65 days (range 25–316 days). Due to uncertainty about the pre-operative diagnosis, resection was attempted in 25 patients (26.3%). Gross total resection was completed in 13 patients (13.7%) based on the operative report and no contrast-enhancing disease on the post-operative MRI. Of these, four patients opted for surveillance instead of additional therapy. The remaining 12 patients (12.6%) had sub-total resection. In total, 35 patients developed disease progression. Among them, WBRT was used in the salvage setting for 12 patients (34.3%), salvage systemic therapy was used for 18 patients (51.4%), and intrathecal therapy was used for 5 patients (14.3%). Demographic and treatment characteristics for the subgroups treated with systemic therapy or with WBRT are summarized in Table 1.

All of the patients who received first-line systemic therapy completed at least one cycle of HD-MTX. The median dose of the first cycle of MTX was 7.4 g/m^2^ (range 2.7–8.3 g/m^2^), and 33 patients (53.2%) completed at least six cycles. HD-MTX alone was used for 19 patients (30.6%), while 21 patients (33.9%) received RMT [18] and 17 patients (27.4%) received HD-MTX and rituximab alone. Additionally, one patient was treated with MPV (HD-MTX, vincristine, and procarbazine), one patient was treated with MATRix (HD-MTX, cytarabine, thiotepa, and rituximab), two patients were treated with HD-MTX and cytarabine, and one patient had an autologous stem cell transplant after the induction HD-MTX. When rituximab was used, it was at a dose of 375 mg/m^2^, delivered via IV twice per cycle, and scheduled to be given with every cycle of HD-MTX.

Intrathecal chemotherapy was administered to 12 patients, who were also treated with systemic therapy (19.4%). Intrathecal chemotherapy was used for five patients with positive cytology, two patients with suspected spinal dissemination based on symptoms with inconclusive radiographic findings and negative cytology, two patients with negative cytology but who initiated treatment at an outside facility, and three patients with no evaluable CSF prior to treatment initiation.

### 3.3. Associations with Patient Outcomes

**Figure 2:**
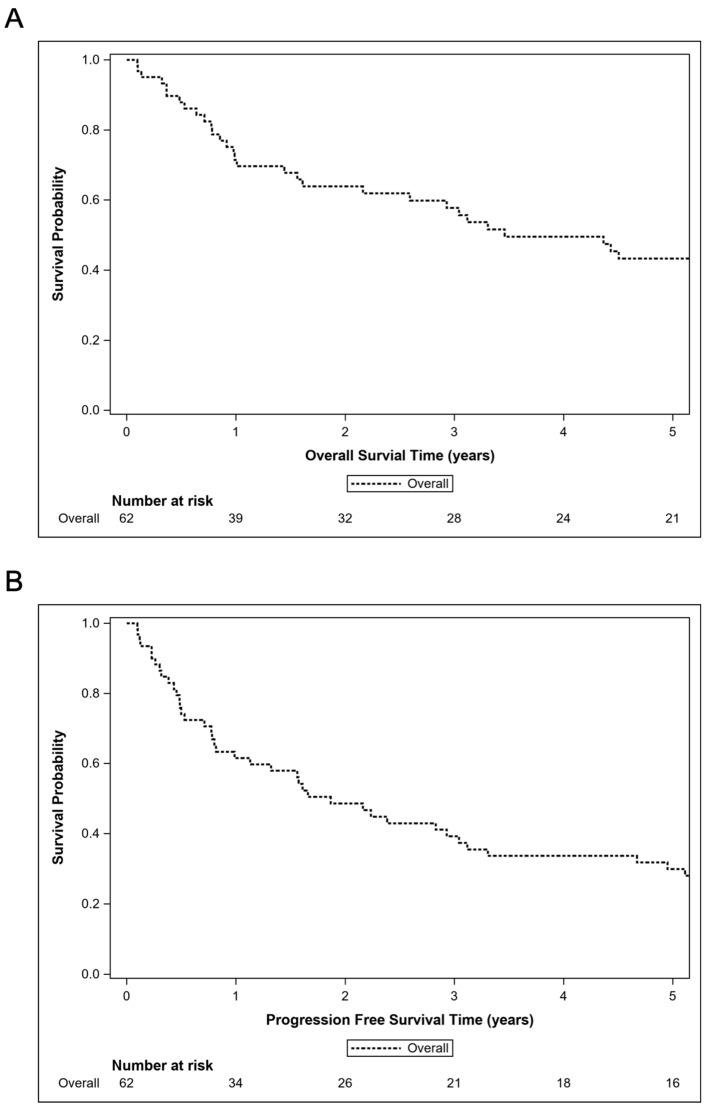
Kaplan–Meier curves for the cohort of patients treated with systemic therapy, depicting the 5-year overall survival (**A**) and progression-free survival (**B**).

**Figure 3:**
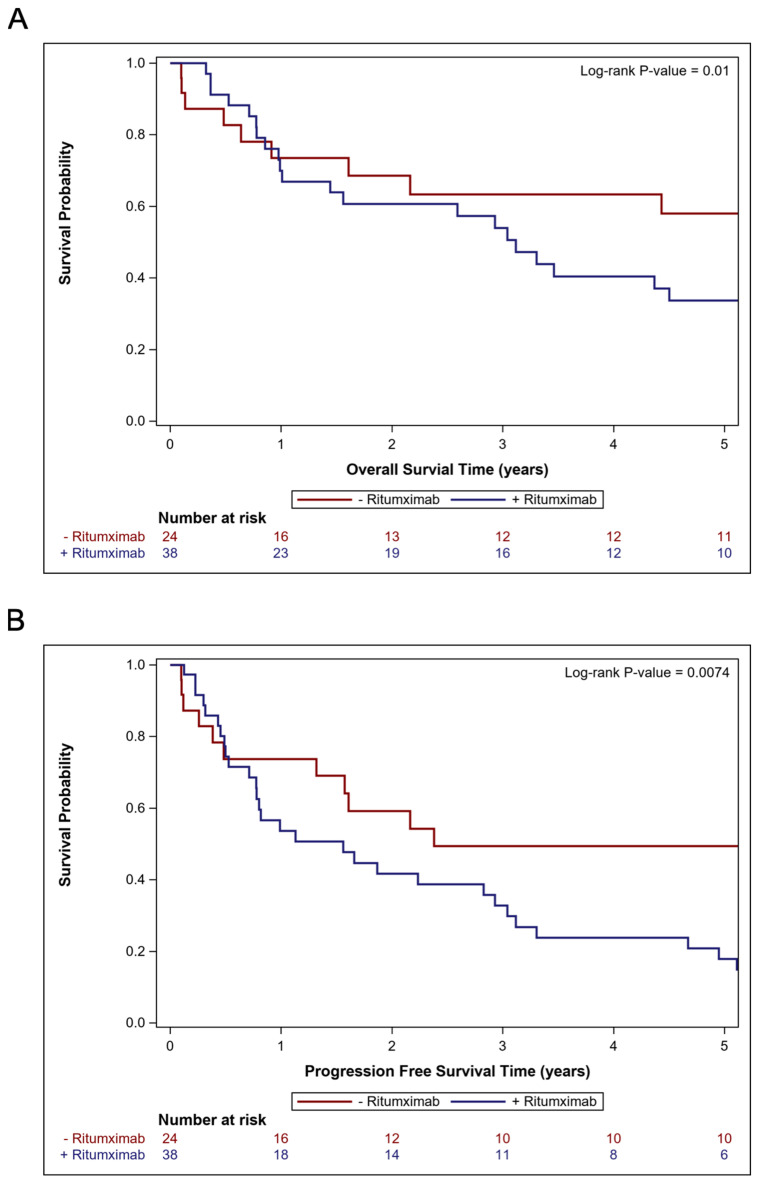
Kaplan–Meier curves for patients treated with systemic therapy with or without rituximab, depicting the 5-year overall survival (**A**) and progression-free survival (**B**).

For all 95 patients, the median OS was 2.2 years (95% CI: 0.9–3.6 years) and the 2-year OS rate was 50.1% (95% CI: 38.6–60.5%) after a median follow-up of 8.9 months (range 0.2–210.3 months). Amongst the 62 patients treated with systemic therapy, the median OS was 3.5 years (95% CI: 1.6–5.7 years) and the 2-year OS rate was 63.9% (95% CI: 49.8–75.0%). The Kaplan–Meier curves for the OS of patients treated with systemic therapy are shown in Figure 2A. The UVA and MVA analyses for the OS and PFS for patients treated with upfront systemic therapy are summarized in Table 2. On UVA, better OS was significantly associated with a younger age (*p* = 0.020), patients who were not solid organ transplant recipients (*p* = 0.029), the completion of at least six cycles of HD-MTX (*p* = 0.005), and the use of systemic therapy without rituximab (*p* = 0.012). On MVA, better OS was associated with the completion of at least six cycles of HD-MTX (median OS 5.4 years vs. 1.0 years; HR 0.40; 95% CI: 0.21–0.78; *p* = 0.007) and the use of systemic therapy without rituximab compared to with rituximab (median OS 7.1 years vs. 3.1 years; HR 2.82; 95% CI: 1.37–5.83; *p* = 0.005). The Kaplan–Meier curves for the OS of patients treated with systemic therapy stratified by rituximab use are shown in Figure 3A. The Kaplan–Meier curves for the OS of patients treated with WBRT are shown in Figure S1A.

In the entire cohort, the median PFS was 0.9 years (95% CI: 0.5–1.9 years) and the 2-year PFS rate was 38.5% (95% CI: 27.9–49.0%). Amongst the 62 patients treated with systemic therapy, the median PFS was 1.9 years (95% CI: 0.8–3.0 years) and the 2-year PFS rate was 48.6% (95% CI: 35.0–60.9%). The Kaplan–Meier curves for the PFS of patients treated with systemic therapy are shown in Figure 2B. On UVA (Table 2), better PFS was significantly associated with a younger age (*p* = 0.070), patients who were not solid organ transplant recipients (*p* = 0.027), the completion of at least six cycles of HD-MTX (*p* = 0.006), and the use of systemic therapy without rituximab (*p* = 0.009). On MVA (Table 2), better PFS was associated with the completion of at least six cycles of HD-MTX (*p* = 0.007) and the use of systemic therapy without rituximab (*p* = 0.008). The Kaplan–Meier curves for the PFS of patients treated with systemic therapy stratified by rituximab use is shown in Figure 3B. The UVA and MVA results are summarized in Table 2. The Kaplan–Meier curves for the PFS of patients treated with WBRT are shown in Figure S1B.

### 3.4. Comparisons of Cohorts Treated with and Without Rituximab

Due to the association between rituximab use and a shorter OS and PFS, we performed post hoc exploratory analyses. Patients treated with rituximab were significantly more likely to be treated with temozolomide (55.3% vs. 0%; *p* < 0.001). Comparing patients treated with different systemic therapy regimens, there were no significant baseline differences in age, KPS, HIV or solid organ transplant status, histology, the number or size of the initial tumor burden, the proportion of patients with an initial positive CSF cytology, the proportion of patients completing at least six cycles of HD-MTX, the receipt of upfront resection, or WBRT use (Table 3). When assessing systemic therapy regimens instead of the inclusion of individual agents, HD-MTX alone had a significantly lower hazard for death (HR 0.37; 95% CI: 0.16–0.86; *p* = 0.021) and progression or death (HR 0.37; 95% CI: 0.17–0.80; *p* = 0.011) on UVA compared to RMT (Table S1). There was no significant difference between RMT and HD-MTX with rituximab on UVA for the OS (HR 0.99; 95% CI 0.46–2.10; *p* = 0.974) or PFS (HR 0.84; 95% CI: 0.41–1.69; *p* = 0.618). However, on MVA, when also incorporating the year of diagnosis, the systemic therapy regimen was not significantly associated with OS or PFS, but the year of diagnosis was significant for the OS (HR 1.26; 95% CI: 1.14–1.39; *p* < 0.001) and PFS (HR 1.18; 95% CI 1.09–1.28; *p* < 0.001).

## 4. Discussion

In this large single-institution cohort of patients with PCNSL, the completion of at least six cycles of HD-MTX was associated with improved disease control and overall survival, but worse outcomes were associated with the use of rituximab. Apart from a systemic therapy regimen, there were no significant differences between patient or disease characteristics between the patients in our cohort who did or did not receive rituximab. These real-world data parallel the randomized trial data showing no significant improvement in outcomes with the incorporation of rituximab into the management of PCNSL [12,15]. Throughout the study period, the long-term survival remained low, underscoring the continued unmet needs for these patients.

While rituximab is endorsed by the NCCN for PCNSL [6], and has a significant benefit in the treatment of systemic lymphoma, the role of rituximab incorporated into induction therapy in PCNSL is less clear [12,15]. Early results from E1F05, a single-arm Phase II trial from ECOG-ACRIN, showed better survival with HD-MTX, vincristine, procarbazine, cytarabine, dexamethasone, and rituximab compared to historical controls on RTOG 93–10 treated with HD-MTX, vincristine, and procarbazine [19]. Furthermore, in IELSG32, a larger multi-stage trial, patients treated with HD-MTX, cytarabine, and rituximab had numerically, but not significantly, better PFS (*p* = 0.051) and OS (*p* = 0.095) compared to HD-MTX and cytarabine [12]. However, in the HOVON 105/ALLG NHL 24 trial, patients treated with HD-MTX, carmustine, teniposide, and prednisone with or without rituximab had no difference in event-free survival (*p* = 0.99) [15]. In our cohort, systemic therapy intensification with rituximab—often in tandem with the addition of temozolomide—was the most common regimen, with no discernable pattern for use or omission. Notably, JCOG1114C showed that the addition of temozolomide to a backbone of WBRT and HD-MTX was closed early due to futility, with numerically worse OS in the temozolomide arm (HR 2.18; 95% CI: 0.95–4.98) [20]. Similarly, patients in our study treated with more intensive systemic therapy using rituximab with or without temozolomide had significantly worse OS and PFS. Further work should examine real-world data to determine if the cytopenias correlate with incomplete treatment, infections, and treatment delays, or with earlier recurrences.

Our study also reaffirmed HD-MTX as the backbone of therapy for PCNSL. In this cohort, we showed that the completion of the induction of HD-MTX is critically important for OS and PFS, which is consistent with the international consensus [6,21,22,23,24]. However, the optimal adjunctive or consolidative therapy in combination with HD-MTX has remained elusive. Multiple systemic therapies have been added to the HD-MTX backbone, including rituximab [12], temozolomide [10], stem cell transplants [11], as well as other combinations [8,9]. While non-CNS lymphoma may benefit from a combination of radiotherapy and systemic therapies [25], WBRT is less routinely used in PCNSL due to data reporting that combining HD-MTX with WBRT can lead to severe neurotoxicity in as many as 49% of patients, which is almost double the rate of neurotoxicity as HD-MTX alone [7]. The overall poor OS and PFS in our series and other reports, however, highlights an urgent need for new and tolerable systemic therapies. Ongoing trials are investigating new agents for induction therapy, such as lenalidomide, ibrutinib, or voraxaze [26,27]. Immune checkpoint inhibitors, which have dramatically improved outcomes for metastatic solid malignancies, are also being investigated for relapsed or refractory PCNSL [28,29,30,31].

Intrathecal therapies may benefit select patients, but are typically reserved for patients with disseminated disease, as data are sparse in patients with localized disease [32,33]. In a small study of 26 patients, in which 46% of the patients had localized disease, continuous-infusion intrathecal MTX with WBRT, with or without systemic HD-MTX, resulted in median PFS and OS of 59.4 months and 93.8 months. These authors postulated that the favorable results may be due to the more stable MTX concentration in the CSF [34]. Similarly, a Phase II study without WBRT showed a 3-year OS and PFS of 84.2% and 63.2% with systemic rituximab, idarubicin, dexamethasone, cytarabine, and MTX plus intrathecal rituximab, dexamethasone, cytarabine, and MTX [35]. By comparison, the use of rituximab, HD-MTX, procarbazine, and vincristine, followed by reduced-dose WBRT and cytarabine, was shown to prospectively have a 3-year OS of 87% and a 2-year PFS of 77% [36]. The combination of HD-MTX and IT MTX, however, comes at the expense of severe toxicity, including Grade 5 adverse events [37]. In our study, we observed numerically, albeit not statistically, significantly superior OS and PFS with intrathecal therapy among a subset of 12 patients. Further investigation into whether intrathecal therapies may improve outcomes is warranted, particularly among patients ineligible for systemic HD-MTX.

This retrospective, single-institution study has multiple limitations. A variety of treatment approaches with varying drug combinations were used, which limits our ability to evaluate the impact of each variable independently, and provider biases may have unknown confounding effects. However, this limitation does allow us to examine differing treatments in the same hospital system over time. Follow-up is limited, and given the aggressive nature of PCNSL, our outcomes may be an over-estimate due to censoring. Additionally, the limited sample size could result in Type II errors regarding the impact of the initial tumor burden, temozolomide, WBRT, or GTR. The demographics and outcomes of patients treated at a single center in the United States may not generalize to other regions, particularly as the use of stem cell transplant and consolidative WBRT was relatively low. Notably, many patients with PCNSL present with poor performance status, and real-world data may more accurately reflect their prognosis than that achieved in clinical trials.

## 5. Conclusions

This study reaffirms the importance of HD-MTX and questions the benefit of rituximab for the induction treatment of PCNSL. The outcomes remain poor, and further work is needed to optimize systemic therapies in the upfront and relapsed settings. Bulky disease was associated with numerically worse outcomes, but there were no associations with the benefits of radiotherapy or aggressive surgery. Factors that predict the potential benefit of non-systemic therapies may help to improve outcomes for subgroups of patients with this rare disease.

## Figures and Tables

**Table 1:** Patient, tumor, and treatment characteristics for the entire cohort, the cohort treated with systemic therapy, and the cohort treated with whole-brain radiotherapy alone (WBRT).

Variable	Level	Overall Cohort (*n* = 95)	Systemic Therapy Cohort (*n* = 62)	WBRT Cohort (*n* = 13)
Age	≥65	36 (37.9%)	25 (40.3%)	3 (23.1%)
	<65	59 (62.1%)	37 (59.7%)	10 (76.9%)
KPS	≥70	61 (64.2%)	44 (71.0%)	4 (30.8%)
	<70	34 (35.8%)	18 (29.0%)	9 (69.2%)
HIV	Positive	10 (10.5%)	1 (1.6%)	5 (38.5%)
	Negative	85 (89.5%)	61 (98.4%)	8 (61.5%)
Solid Organ Transplant	Yes	4 (4.2%)	1 (1.6%)	3 (23.1%)
	No	91 (95.8%)	61 (98.4%)	10 (76.9%)
Initial Number of Lesions	2+	36 (51.4%)	26 (51.0%)	3 (50.0%)
	1	34 (48.6%)	25 (49.0%)	3 (50.0%)
	Missing	25	1	7
Initial Size of Lesions	≥14 cc	32 (45.7%)	23 (45.1%)	2 (33.3%)
	<14 cc	38 (54.3%)	28 (54.9%)	4 (66.7%)
	Missing	25	11	7
Histology	DLBCL	87 (91.6%)	56 (90.3%)	13 (100.0)
	Marginal Zone	5 (5.3%)	3 (4.8%)	0 (0.0%)
	T-Cell	3 (3.2%)	3 (4.8%)	0 (0.0%)
CSF Cytology	Positive	18 (30.0%)	18 (39.1%)	0 (0.0%)
	Negative	42 (70.0%)	28 (60.9%)	5 (100.0%)
	Missing	35	16	8
Cycles of HD-MTX	≥6	33 (34.7%)	35 (56.5%)	0 (0.0%)
	0–5	62 (65.3%)	27 (43.5%)	13 (100.0%)
Upfront Rituximab	Yes	38 (61.3%)	38 (61.3%)	0 (0.0%)
	No	24 (38.7%)	24 (38.7%)	13 (100.0%)
	Missing	33	0	0
Upfront Temozolomide	Yes	21 (33.9%)	41 (66.1%)	0 (0.0%)
	No	41 (66.1%)	21 (33.9%)	13 (100.0%)
	Missing	33	0	0
Intrathecal Chemotherapy	Yes	12 (19.4%)	49 (79.0%)	0 (0.0%)
	No	50 (80.6%)	13 (21.0%)	13 (100.0%)
	Missing	33	0	0
Type of Surgery	Biopsy	70 (73.7%)	49 (79.0%)	12 (92.3%)
	GTR	13 (13.7%)	6 (9.7%)	0 (0.0%)
	STR	12 (12.6%)	7 (11.3%)	1 (7.7%)
Radiotherapy Use	None	54 (65.9%)	47 (75.8%)	0 (0.0%)
	First-Line	16 (19.5%)	3 (4.8%)	13 (100.0%)
	Salvage	12 (14.6%)	12 (19.4%)	0 (0.0%)
	Missing	13	0	0

First-line radiotherapy either as monotherapy or consolidation after chemotherapy. Missing for rituximab or temozolomide either for patients not treated with upfront chemotherapy or with an unknown systemic therapy used.

**Table 2:** Univariate and multivariable analyses of the associations between patient, tumor, and treatment characteristics, with the overall survival and progression-free survival amongst patients treated with systemic therapy.

			Overall Survival				Progression-Free Survival			
			UVA		MVA		UVA		MVA	
Variable	Level	*n*	Hazard Ratio (95% CI)	*p*-Value	Hazard Ratio (95% CI)	*p*-Value	Hazard Ratio (95% CI)	*p*-Value	Hazard Ratio (95% CI)	*p*-Value
Age	≥65	25	2.11 (1.12–3.95)	0.020	1.89 (0.98–3.62)	0.057	1.68 (0.96–2.96)	0.070	1.51 (0.85–2.68)	0.160
	<65	37	-	-	-	-	-	-	-	-
KPS	≥70	18	-	-			-	-		
	<70	44	1.39 (0.69–2.78)	0.356			1.26 (0.66–2.39)	0.488		
HIV	Positive	1	1.09 (0.15–7.99)	0.935			0.88 (0.12–6.46)	0.903		
	Negative	61	-	-			-	-		
Solid Organ Transplant	Yes	1	10.98 (1.28–94.08)	0.029			11.34 (1.32–97.09)	0.027		
	No	61	-	-			-	-		
Initial Number of Lesions	2+	26	1.36 (0.71–2.59)	0.349			1.26 (0.70–2.26)	0.442		
	1	25	-	-			-	-		
Initial Size of Lesions	≥14 cc	23	1.30 (0.69–2.44)	0.418			1.09 (0.60–1.99)	0.766		
	<14 cc	28	-	-			-	-		
CSF Cytology	Positive	18	-	-			-	-		
	Negative	28	1.19 (0.59–2.41)	0.624			1.07 (0.56–2.04)	0.847		
Cycles of HD-MTX	≥6	35	0.40 (0.21–0.76)	0.005	0.40 (0.21–0.78)	0.007	0.44 (0.24–0.79)	0.006	0.44 (0.24–0.80)	0.007
	0–5	27	-	-	-	-	-	-	-	-
Upfront Rituximab	Yes	38	2.46 (1.22–4.97)	0.012	2.82 (1.37–5.83)	0.005	2.31 (1.24–4.33)	0.009	2.37 (1.26–4.48)	0.008
	No	24	-	-	-	-	-	-	-	-
Upfront Temozolomide	Yes	21	1.68 (0.87–3.24)	0.120			1.77 (0.96–3.27)	0.069		
	No	41	-	-			-	-		
Intrathecal Chemotherapy	Yes	13	0.59 (0.27–1.27)	0.174			0.68 (0.35–1.33)	0.262		
	No	49	-	-			-	-		
Type of Surgery	Biopsy	49	1.78 (0.72–4.40)	0.213			2.15 (0.89–5.19)	0.09		
	GTR	6	1.85 (0.55–6.28)	0.323			2.62 (0.81–8.44)	0.107		
	STR	7	-	-			-	-		
Consolidation Radiotherapy	No	59	-	-			-	-		
	Yes	3	0.24 (0.03–1.74)	0.156			0.37 (0.09–1.59)	0.182		

**Table 3:** Patient, tumor, and treatment characteristics for the cohorts treated with upfront chemotherapy high-dose methotrexate (HD-MTX) alone, HD-MTX and rituximab, or HD-MTX, rituximab, and temozolomide (RMT); *p*-values for the univariate differences between the cohorts.

Variable	Level	RMT Regimen (*n* = 21)	HD-MTX and Rituximab (*n* = 17)	HD-MTX Alone (*n* = 19)	*p*-Value
Age	≥65	11 (52.4%)	10 (58.8%)	12 (63.2%)	0.785
	<65	10 (47.6%)	7 (41.2%)	7 (36.8%)	
KPS	≥70	13 (61.9%)	12 (70.6%)	15 (78.9%)	0.500
	<70	8 (38.1%)	5 (29.4%)	4 (21.1%)	
HIV	Positive	0 (0.0%)	1 (5.9%)	0 (0.0%)	0.302
	Negative	21 (100.0%)	16 (94.1%)	19 (100.0%)	
Solid Organ Transplant	Yes	0 (0.0%)	1 (5.9%)	0 (0.0%)	0.302
	No	21 (100.0%)	16 (94.1%)	19 (100.0%)	
Initial Number of Lesions	2+	12 (60.0%)	6 (37.5%)	8 (61.5%)	0.314
	1	8 (40.0%)	10 (62.5%)	5 (38.5%)	
	Missing	1	1	6	
Initial Size of Lesions	≥14 cc	9 (45.0%)	8 (50.0%)	5 (38.5%)	0.824
	<14 cc	11 (55.0%)	8 (50.0%)	8 (61.5%)	
	Missing	1	1	6	
Histology	DLBCL	20 (95.2%)	16 (94.1%)	16 (84.2%)	0.383
	Marginal Zone	1 (4.8%)	1 (5.9%)	1 (5.3%)	
	T-Cell	0 (0.0%)	0 (0.0%)	2 (10.5%)	
CSF Cytology	Positive	8 (42.1%)	2 (18.2%)	6 (46.2%)	0.310
	Negative	11 (57.9%)	9 (81.8%)	7 (53.8%)	
	Missing	2	6	6	
Cycles of HD-MTX	≥6	15 (71.4%)	7 (41.2%)	11 (57.9%)	0.171
	0–5	6 (28.6%)	10 (58.8%)	8 (42.1%)	
Upfront Rituximab	Yes	21 (100.0%)	17 (100.0%)	0 (0.0%)	<0.001
	No	0 (0.0%)	0 (0.0%)	0 (0.0%)	
Upfront Temozolomide	Yes	21 (100.0%)	0 (0.0%)	0 (0.0%)	<0.001
	No	0 (0.0%)	0 (0.0%)	0 (0.0%)	
Intrathecal Chemotherapy	Yes	1 (4.8%)	4 (23.5%)	3 (15.8%)	0.245
	No	20 (95.2%)	13 (76.5%)	16 (84.2%)	
Type of Surgery	Biopsy	16 (76.2%)	13 (76.5%)	15 (78.9%)	0.888
	GTR	3 (14.3%)	2 (11.8%)	1 (5.3%)	
	STR	2 (9.5%)	2 (11.8%)	3 (15.8%)	
Radiotherapy Use	None	15 (71.4%)	13 (76.5%)	15 (78.9%)	0.418
	First Line	0 (0.0%)	1 (5.9%)	2 (10.5%)	
	Salvage	6 (28.6%)	3 (17.6%)	2 (10.5%)	

## Data Availability

Data will be made available upon reasonable request to the corresponding author.

## Supplementary Materials

The following supporting information can be downloaded at: https://www.mdpi.com/article/10.3390/cancers17040618/s1, Figure S1: Kaplan–Meier curves for patients treated with whole-brain radiotherapy, depicting the 5-year overall survival (A) and progression-free survival (B); Table S1: Univariate and multivariable analyses of associations between patient, tumor, and treatment characteristics, with the overall survival and progression-free survival amongst patients treated with systemic therapy.
