# Supplementary material for: Impact of Systemic and Radiation Therapy on Survival of Primary Central Nervous System Lymphoma
Source: Cancers (Basel). 2025 Feb 12;17(4):618. doi: 10.3390/cancers17040618 (PMC11853249; doi:10.3390/cancers17040618)
Supplement: Supplementary file 1 [file cancers-17-00618-s001.zip › Supplemental Table S1.pdf]

**Supplemental Table S1**: Univariate and Multivariable analyses of associations between patient, tumor, and treatment characteristics with overall survival and progression free survival amongst patients treated with systemic therapy.

| | | | Overall Survival | | | | Progression Free Survival | | | |
|---|---|---|---|---|---|---|---|---|---|---|
| | | | UVA | | MVA | | UVA | | MVA | |
| Variable | Level | n | Hazard Ratio (95% CI) | P-value | Hazard Ratio (95% CI) | P-value | Hazard Ratio (95% CI) | P-value | Hazard Ratio (95% CI) | P-value |
| Year of Diagnosis | Continuous | 62 | 1.26 (1.15-1.39) | <0.001 | 1.26 (1.14-1.39) | <0.001 | 1.15 (1.07-1.23) | <0.001 | 1.18 (1.09-1.28) | <0.001 |
| Age | ≥65 | 25 | 2.11 (1.12-3.95) | 0.020 | | | 1.68 (0.96-2.96) | 0.070 | 1.10 (0.61-2.00) | 0.746 |
| | <65 | 37 | - | - | | | - | - | - | - |
| KPS | ≥70 | 18 | - | - | | | - | - | | |
| | <70 | 44 | 1.39 (0.69-2.78) | 0.356 | | | 1.26 (0.66-2.39) | 0.488 | | |
| HIV | positive | 1 | 1.09 (0.15-7.99) | 0.935 | | | 0.88 (0.12-6.46) | 0.903 | | |
| | negative | 61 | - | - | | | - | - | | |
| Solid Organ Transplant | Yes | 1 | 10.98 (1.28-94.08) | 0.029 | | | 11.34 (1.32-97.09) | 0.027 | | |
| | No | 61 | - | - | | | - | - | | |
| Initial Number of Lesions | 2+ | 26 | 1.36 (0.71-2.59) | 0.349 | | | 1.26 (0.70-2.26) | 0.442 | | |
| | 1 | 25 | - | - | | | - | - | | |
| Initial Size of Lesions | ≥ 14 cc | 23 | 1.30 (0.69-2.44) | 0.418 | | | 1.09 (0.60-1.99) | 0.766 | | |
| | < 14 cc | 28 | - | - | | | - | - | | |
| CSF Cytology | Positive | 18 | - | - | | | - | - | | |
| | Negative | 28 | 1.19 (0.59-2.41) | 0.624 | | | 1.07 (0.56-2.04) | 0.847 | | |
| Cycles of HD-MTX | ≥6 | 35 | 0.40 (0.21-0.76) | 0.005 | 0.46 (0.24-0.88) | 0.018 | 0.44 (0.24-0.79) | 0.006 | 0.45 (0.24-0.85) | 0.013 |
| | 0-5 | 27 | - | - | - | - | - | - | - | - |
| Type of Chemotherapy | HD-MTX alone | 19 | 0.37 (0.16-0.86) | 0.021 | | | 0.37 (0.17-0.80) | 0.011 | | |
| | HD-MTX and Rituximab | 17 | 0.99 (0.46-2.10) | 0.974 | | | 0.84 (0.41-1.69) | 0.618 | | |
| | RMT | 21 | - | - | | | - | - | | |

| | | | | | | |
|---|---|---|---|---|---|---|
| Intrathecal Chemotherapy | Yes | 12 | 0.59 (0.27-1.27) | 0.174 | 0.68 (0.35-1.33) | 0.262 |
| | No | 49 | - | - | - | - |
| Type of Surgery | Biopsy | 49 | 1.78 (0.72-4.40) | 0.213 | 2.15 (0.89-5.19) | 0.09 |
| | GTR | 6 | 1.85 (0.55-6.28) | 0.323 | 2.62 (0.81-8.44) | 0.107 |
| | STR | 7 | - | - | - | - |
| Consolidation Radiotherapy | No | 59 | - | - | - | - |
| | Yes | 3 | 0.24 (0.03-1.74) | 0.156 | 0.37 (0.09-1.59) | 0.182 |

RMT stands for rituximab, temozolomide, and HD-MTX.
