# Supplementary figures and images for: Impact of Systemic and Radiation Therapy on Survival of Primary Central Nervous System Lymphoma
Source: Cancers (Basel). 2025 Feb 12;17(4):618. doi: 10.3390/cancers17040618 (PMC11853249; doi:10.3390/cancers17040618)

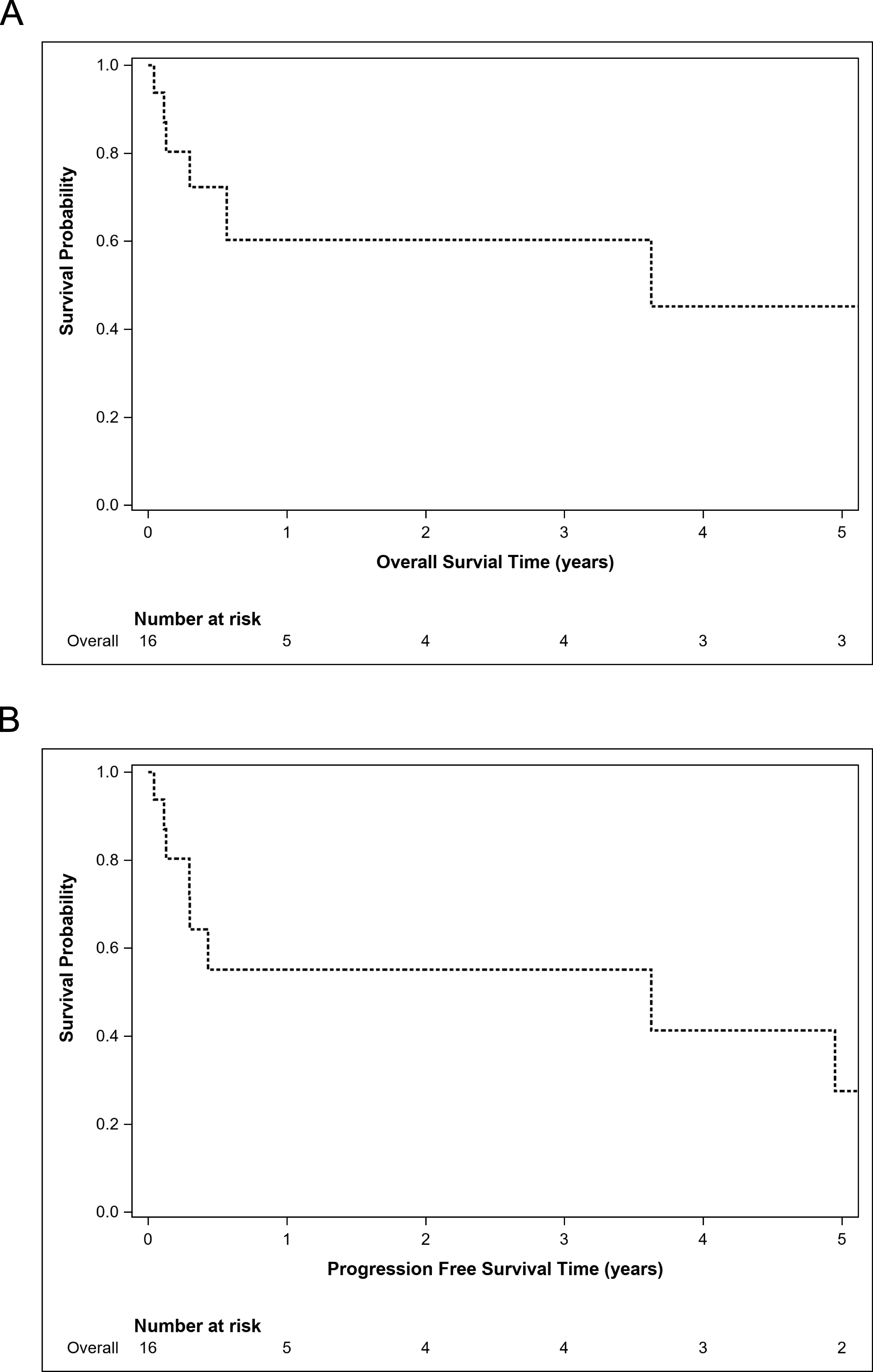

Supplement: Supplementary file 1 [file cancers-17-00618-s001.zip › Supplemental Figure S1.tiff]
